# Rasterstereographic Analysis of Lateral Shift in Patients with Lumbar Disc Herniation: A Case Control Study

**DOI:** 10.1155/2018/6567139

**Published:** 2018-10-01

**Authors:** Britta K. Krautwurst, Jürgen R. Paletta, Sylvia Mendoza, Adrian Skwara, Melvin Mohokum

**Affiliations:** ^1^Heidelberg University Clinics, Department for Orthopedics and Trauma Surgery, Schlierbacher Landstraße 200a, 66118 Heidelberg, Germany; ^2^Department of Orthopedics and Rheumatology, University Hospital Marburg, Baldingerstrasse, 35043 Marburg, Germany; ^3^Studiendekanat, Fachbereich Medizin, Dr. Reinfried Pohl-Zentrum für Medizinische Lehre (RPZ), University Hospital Marburg, Conradistraße 9, 35043 Marburg, Germany; ^4^Orthopädische Gemeinschaftspraxis Ammenwerth / Skwara, Schildern 6, 33098 Paderborn, Germany; ^5^SRH Hochschule für Gesundheit, University of Applied Health Science, Neue Str. 28–30, 07548 Gera, Germany

## Abstract

**Objective:**

Detection of a lateral shift (LS) in patients with diagnosed disc herniation compared to healthy controls.

**Summary of Background Data:**

A specific lateral shift (LS) pattern is observed in patients with disc herniation and low back pain, as shown in earlier studies.

**Methods:**

Rasterstereography (RS) was used to investigate the LS. Thirty-nine patients with lumbar disc herniation diagnosed by radiological assessment and low back pain and/or leg pain (mean age 48.2 years, mean BMI 28.5, 28 males and 11 females) and 36 healthy controls (mean age 47.4 years, mean BMI 25.7, 25 males and 11 females) were analysed. LS, pelvic tilt, pelvic inclination, lordotic angle, and trunk torsion were assessed.

**Results:**

The patient group showed a nonsignificant increase in LS, that is, 5.6 mm compared to the healthy controls with 5.0 mm (*p* = 0.693). However, significant differences were found between groups regarding pelvic tilt in degrees (patients 5.9°, healthy controls 2.0°;* p* = 0.016), trunk torsion (patients 7.5°, controls 4.5°;* p* = 0.017), and lordotic angle (patients 27.5°, healthy controls 32.7°;* p* = 0.022). The correlation between pain intensity and the FFbH-R amounted 0.804 (p = < 0.01), and that between pain intensity and the pain disability index was 0.785 (*p* < 0.01).

**Discussion:**

Although some studies have illustrated LS with disc herniation and low back pain, the present findings demonstrate no significant increase in LS in the patient group compared to healthy controls.

**Conclusion:**

The patients with lumbar disc herniation did not demonstrate an increased LS compared to healthy controls. Other parameters like pelvic tilt and inclination seemed to be more suitable to identify changes in posture measured by RS in patients with low back pain or disc herniation.

## 1. Introduction

Low back pain is currently a common problem with a considerable medical and therapeutic impact [1]. The lifetime prevalence amounts up to 80% and higher in the general population [2–4]. In approximately 85% of cases, the underlying cause is unknown, while in around 15%, the cause is known, for example, lumbar disc herniation [5]. Lumbar disc herniation has a high prevalence and affects the spine in younger and middle-aged patients [6–9]. For clinical decision making and to initiate a specific and appropriate therapy for the patient group, a systematic investigation of patients with lumbar disc herniation and low back pain is required. One parameter that can be assessed is the lateral shift (LS), which is an important clinical sign. It occurs ipsilaterally or contralaterally, with no relation to the side of pain [10]. There are different definitions available for the LS [11]. A LS is a deviation from the spinal midline. This is shown by the sagittal arrangement of the lumbar spinous processes. They are typically arranged asymmetrically. The upper body is clearly shifted to the side [11]. The LS can be determined with different methods, for example, visual investigation using a plumbline and palpation using the side-glide test sequence [12, 13]. The LS can be determined with an accuracy of 5 mm using the plumbline technique [14]. The prevalence of LS in patients with low back pain has been previously examined [10, 12]. In a collective of patients with an acute episode of low back pain, Gillan showed an abnormality in LS ranging from 5 to 50 mm and called it a “new phenomenon associated with the onset of back pain” [12]. Porter et al. examined 100 patients with back pain and LS; the majority (71 patients) reported pain distribution below the knee [10]. Healthy controls were not evaluated.

One measurement method that illustrates important spine parameters is rasterstereography [15]. RS is a contact-free, noninvasive technique to detect spinal deformities, for instance, scoliosis, on the basis of surface asymmetry. The validity and reliability of this method have been described elsewhere [16–22].

Khallaf examined 16 patients with lumbar disc herniation using rasterstereography [23]. The patients showed a significantly increased lateral pelvic tilt and an increased lordotic angle compared to healthy subjects. However, the lateral shift as an important clinical sign is not evaluated by Khallaf. Therefore, the primary aim of this study was to evaluate LS in patients with diagnosed lumbar disc herniation compared to healthy controls measured by RS. The secondary aim was to assess posture modifications in relation to spine, pelvic, and functional parameters. These parameters should be correlated to each other. According to the main aim of the study, the null hypothesis (H0) is that patients with lumbar disc herniation will have no LS compared to healthy controls. The alternative hypothesis (H1) is that patients with lumbar disc herniation will have a greater LS compared to healthy controls.

## 2. Methods

### 2.1. Patients and Recruitment Procedure

All participants were recruited in 2011. The individuals in the patient group were enrolled in a rehabilitation hospital with a special section for chronic spinal diseases at Montanus-Klinik Bad Schwalbach, Germany. The healthy individuals in the control group were recruited from the general population and were examined at a second hospital, that is, University Hospital in Marburg, Germany. Both hospitals used the same measurement system.

### 2.2. Patient Group

The inclusion criteria for the patient group were underwritten patient information and informed consent, the ability to speak the German language, the ability to stand free without any assistance, the ability to lay flat on their back, evidence of lumbar disc herniation or protrusion detected by magnetic resonance imaging (MRI), and low back pain and/or leg pain; all stadia of pain (acute, subacute, chronic) were included.

The exclusion criteria for the patient group were age younger than 18 years, cancer, previous spinal surgery, relevant bone degeneration, red flags, pension request, relevant tattoos or scars on the surface of the back, and no pain on a numeric rating scale (NRS = 0).

### 2.3. Control Group

The inclusion criteria for the healthy controls were the ability to speak the German language, no back or leg pain on a numeric rating scale (NRS = 0), the ability to stand free without any assistance, and the ability to lay flat on their back. The exclusion criteria were age younger than 18 years, cancer, previous spine surgery, relevant spinal degeneration, red flags, pension request, relevant tattoos or scars on the back, chronic low back pain, and lumbar low back pain and/or leg pain.

### 2.4. Ethical Aspects

The study received ethical approval from the independent ethics committee of University Hospital Marburg (reference number Az. 22/11). Information about the procedure and risks were included in the patient information. Participants in the patient and control groups had to provide written informed consent.

### 2.5. Methods of Measurement

#### 2.5.1. Self-Assessment Questionnaire

All participants had to complete a general self-assessment questionnaire to provide individual data, for example, age, height, weight, comorbidities, pain anamneses, and a pain chart.

#### 2.5.2. Numeric Rating Scale (NRS)

The Numeric Rating Scale for Pain (NRS) was used to measure pain intensity. The numeric scale has 11 items with “0” representing no pain and “10” representing the worst pain imaginable [25].

#### 2.5.3. Hannover Functional Ability Questionnaire (FFbH-R)

The level of functional ability was measured by the Hannover Functional Ability Questionnaire (FFbH-R). This instrument was developed for patients with musculoskeletal disorders, comprising 12 short self-administered questionnaires on functional capacity in the activities of daily living [26].

#### 2.5.4. Pain Disability Index (PDI)

The PDI is a comprehensive self-administered questionnaire for assessing disability associated with pain. The respondents indicate the amount of perceived pain-related disability in seven different areas of daily living on an 11-point Likert scale with one end point of 0 (no disability) and the other end point set at 10 (maximum disability). The areas are home, social activities, recreational, occupational, sexual functioning, self-care, and life support activities. Higher scores indicate greater disability. The PDI has been shown to have a correlation of r = 0.7-0.9 with the Oswestry Disability Questionnaire in patients with low back pain [27].

#### 2.5.5. Mainz Pain Staging System (MPSS)

The MPSS is classified in three chronification levels. Four axes were considered: temporal aspects of pain, pain distribution, drug intake, and utilisation of health care. The final score described three chronification levels. On level I, the pain is intermittently, temporary, with changeable intensity, mostly in one localization, adequate drug intake, visiting just one medical specialist, and not more than one stay in a hospital due to pain. Level II is characterized as follows: the pain during a longer time, more than one pain localization, drug abuse, changing medical consultations, and 2-3 clinical stays caused by pain. Continuous pain, pain on a large areal and changing localizations, long-time drug abuse, and more than three alterations of the medical specialist and clinical stays describe level III.

#### 2.5.6. Straight Leg Raise (SLR)

The SLR is widely used and is well suited for clinical investigations. The subject lies in a supine position with their head on the ground. The tester lifts the measured leg passively with an extended knee and ankle in the neutral position as high as possible. If the subject indicates pain or the investigator notices resistance, the investigator measures the hip flexion angle at the limit of the SLR using a plurimeter. A positive test result is associated with nerve root compression. The SLR is a reliable tool with a high intraclass reliability of 0.99 [28].

#### 2.5.7. Rasterstereography (RS)

**Figure 1:**
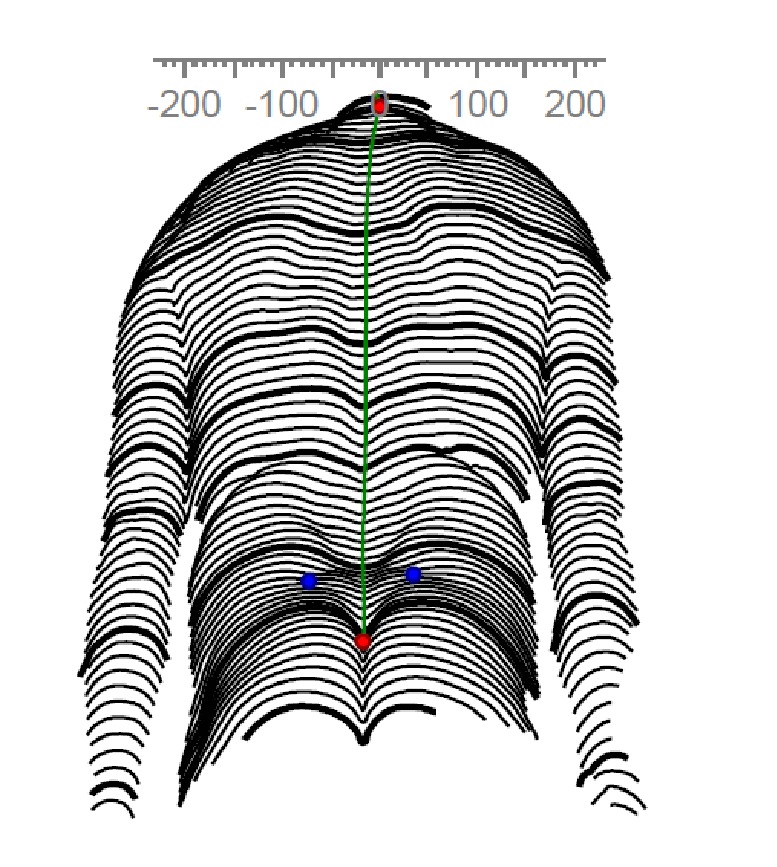
View of the raster lines on a patient's back. Blue dots: left and right lumbar dimples. Lower red dot: dimple mid-point (DM).

RS is a noninvasive technique for analysing back and spinal deformities that uses the triangulation method [29]. Parallel white lines are projected onto the unclothed back of the subject (see Figure 1). This horizontal light raster is detected by a camera system. RS evaluates surface contours formed by the underlying tissue, for example, the spinous process. The system identifies anatomical fixed points with an accuracy of ±0.1 mm standard deviation [30, 31].

The lateral shift is a lateral deviation in the frontal plane. For the calculation the perpendicular from vertebrae prominence was dropped and the difference from this perpendicular to the DM was measured.

**Figure 2:**
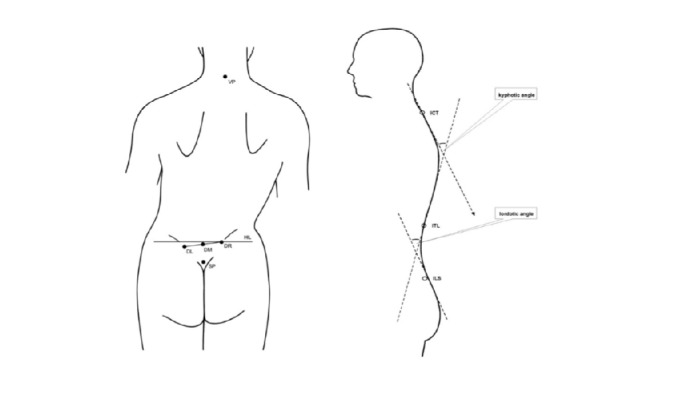
Rasterstereographic parameters: pelvic tilt (left) and lordotic angle (right) adapted by Lippold et al. [24].

**Figure 3:**
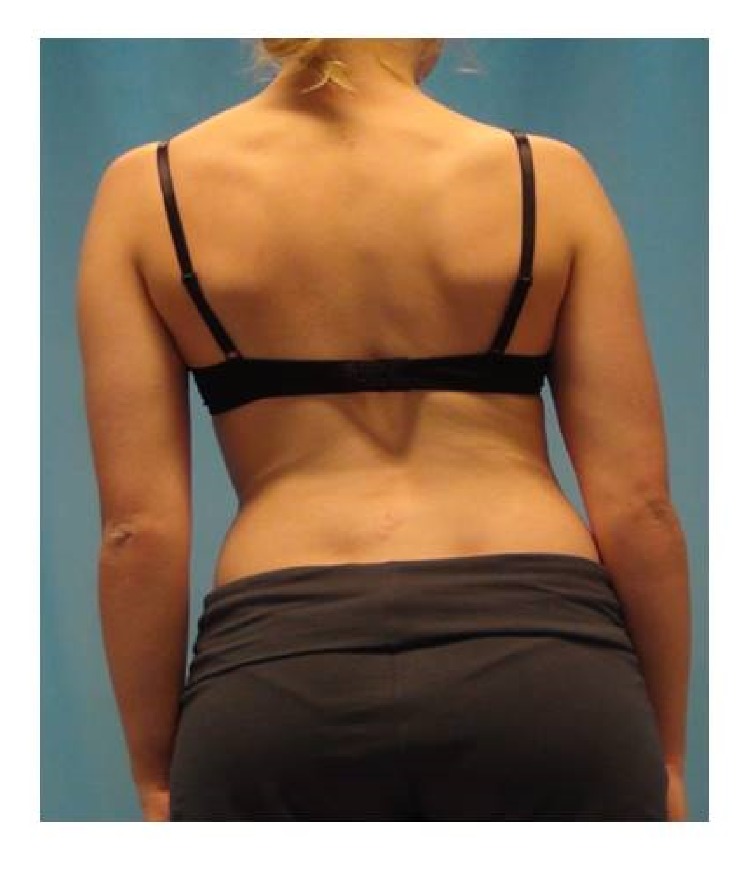
A person with a left LS from the posterior view.

For this study, the following parameters were measured according to Degenhardt 2017 [32]:

(1) Pelvic tilt (PT): Difference in the height of the lumbar dimples is shown in Figure 2 [24]. A positive value indicates a higher right dimple than on the left and a negative value denotes the opposite. This value is quantified in millimetres (mm) and degrees (°).

(2) Pelvic inclination (PI dimples): A positive value indicates the vertical component of the left dimple and the right dimple is adjusted to the top; negative values denote the opposite. This value is provided in degrees.

(3) Pelvic inclination (PI symmetry line): This parameter is a symmetry line of the spinous processes. A positive value indicates an anterior pelvic inclination and a negative value indicates a posterior pelvic inclination. This value is provided in degrees.

(4) Lordotic angle (LA): There are two tangents estimated relating to the surface of the back. The angle between surface tangents of ITL (thoracic-lumbar transition) and ILS (lumbar-sacral transition) is shown in Figure 2 [24]. The higher the value is, the greater the degree of lumbar lordosis is. This value is provided in degrees.

(5) Trunk torsion (TT): This value is provided in degrees.

(6) Lateral shift (LS): This describes the difference in the translative shift (lateral shift) from L1 to DM. This value is provided in degrees. See Figure 3.

#### 2.5.8. Measurement Setup

For this study, the Formetric® III 4 D system (Diers International GmbH, Schlangenbad, Germany) was applied for data collection. It can assess individual clinical parameters under static and dynamic conditions.

To avoid potential bias, the positioning of the subject was standardised. The subjects stood barefoot on a wooden platform with slightly extended knees. The upper extremities hung down lateral to the body, and the subject looked forward. One record took 6 seconds. Two pictures were taken per second. The mean of these 12 pictures of every record was used for the analysis.

#### 2.5.9. Statistical Power Analysis and Statistics

The calculation of the power was based on the data of a pilot study. The difference of LS between healthy subjects and patients was 5.3 mm, the standard deviation 7.9 mm. The significance level was 0.05 and power 0.8. The statistical power analysis suggested a minimal number of 35 subjects for each group (the patients and the healthy controls). A dropout rate of 10% for the sample size was estimated; therefore four additional volunteers were enrolled in each group as a safety margin.

Mean, minimum and maximum values, and standard deviations were calculated for all parameters. The Shapiro-Wilk-Test was used to estimate the normal distribution. The independent sample* t*-test was used to compare the patient and healthy controls. Correlations between the parameters were made using the Pearson correlation coefficient. Differences were accepted as significant when the probability was below 5% (*p* < 0.05). The comparisons of more than one parameter were performed by using one-way ANOVA, with the Bonferroni post hoc test. All statistical analyses were performed with SPSS Version 17 (SPSS Inc., Chicago, IL, USA).

## 3. Results

### 3.1. Characteristics of the Study Participants

In the patient group, 39 patients with a radiologically diagnosed herniated disc were enrolled. Out of the 36 healthy controls, three volunteers had to be excluded: one volunteer reported back pain, and two volunteers had scoliosis. Scoliosis is defined as a deviation of the spine greater than 10° in the coronal plane and an axial rotation [33]. The age range was 25–65 years with a mean age of 48.2 years in the patient group and 47.4 in the control group. 11 women were examined in each group, 28 male patients and 25 healthy men. The characteristics of the participants are shown in Table 1.

### 3.2. Lateral Shift

The lateral shift had a mean value of 5.6±6.0 mm in the patient group and 5.0±7.6 mm in the healthy controls. This difference was not significant (*p* = 0.693). The trunk of the participants with diagnosed disc herniation did not deviate laterally more than the healthy controls.

### 3.3. Lateral Shift in relation to SLR

The participants with a positive SLR demonstrated a slightly increased LS (5.5±5.0°) compared to the participants with negative SLR (5.2±8.1°). However, the difference was not significant (*p* = 0.829).

### 3.4. Trunk, Lumbar Spine, and Pelvis

The patients showed an increased pelvic tilt in [°] as well as [mm], a decreased anterior pelvic inclination (dimples [°] as well as symmetry [°]), an increased trunk torsion [°], and a decreased lordotic angle [°] compared to the healthy controls (Table 2).

### 3.5. Localization of Pain (Pain Chart)

In relation to the localization of pain, there was a significant difference in pelvic tilt in degrees (*p* = 0.036) and pelvic inclination in degrees (*p* = 0.017). The localization of pain has a considerable influence on pelvic inclination (dimples). If pain is not present, the pelvis showed a more anterior tilt (17.8±6.9°) compared to pain up to the feet (11.4±6.6°) (*p* = 0.022).

### 3.6. Correlations between Rasterstereographic Parameters

There was a very high correlation between pelvic tilt [°] and pelvic tilt [mm] of 0.985 (*p* ≤ 0.001) and between pelvic inclination (dimples) [°] and pelvic inclination (symmetry) [°] of 0.904 (*p* ≤ 0.001). A lower correlation was found between trunk torsion [°] and pelvic tilt [°] of 0.390 (*p* ≤ 0.001) and between trunk torsion [°] and pelvic tilt [mm] of 0.382 (*p* ≤ 0.001).

### 3.7. Correlation to FFbH-R

The correlation between FFbH-R and pain intensity was 0.804 (*p* < 0.001), between FFbH-R and BMI 0.310 (*p* = 0.007), between FFbH-R and MPSS 0.445 (*p* = 0.005), between FFbH-R and drug intake 0.545 (*p* < 0.001), and between FFbH-R and PDI 0.793 (*p* < 0.001). A magnitude of 0.643 (*p* < 0.001) revealed the strength of the relationship between FFbH-R and pain chart. See Table 3.

### 3.8. Correlation with the PDI

The correlation between the PDI and weight was 0.246 (*p* = 0.033), between PDI and BMI 0.360 (*p* = 0.002), between PDI and MPSS 0.369 (*p* = 0.021), between PDI and pain chart 0.623 (*p* ≤ 0.001), between PDI and drug intake 0.379 (*p* ≤ 0.001), and between PDI and FFbH-R 0.793 (*p* ≤ 0.001). As the pain intensity increased, then the disability in areas of living increased, too. The magnitude was r = 0.785 (*p* ≤ 0.001). See Table 4.

## 4. Discussion

This study demonstrated that there is no significant LS in patients with diagnosed lumbar disc prolapse compared to healthy controls. Harrison et al. indicated a LS of the thoracic cage relative to the fixed pelvis with digitised anterior-posterior radiographs in a group of healthy volunteers [34]. The displacement was measured from T12 to S2 through a vertical line. The mean value in the left was 53.2±8.4 mm and in the right was 52.1±9.0 mm [34]. Although other studies have illustrated a LS with disc herniation and low back pain, this phenomenon could not be validated in this study. However, other studies did not compare their findings with a control group that included healthy individuals.

This is the first study to evaluate the lateral shift using the rasterstereography. The findings in the present study showed a mean LS in healthy controls of 5.0±7.6 mm and 5.6±6.0 mm in the patient group. This difference was not statistically significant (*p* = 0.693). Other studies did not define LS exactly. A threshold with a precise definition for the presence or absence of LS was also missing. However, in a study by Donahue et al., two therapists indicated a LS range of 1-7 mm in 26 out of 49 patients with low back pain [13]. Harrison and colleagues assessed a spinal rehabilitation program and the effect of LS in patients with chronic low back pain [35]. The control group demonstrated an average LS of 7.2±6.2 mm and an average LS of 15.0±5.9 mm in the treatment group. However, both studies did not compare the results with healthy controls. Donahue et al. reported nonphysiological LS in their patient group [13]. If this phenomenon occurs in healthy subjects, it has not yet been reported. Therefore, in this case-control study, healthy volunteers were included. The LS in the healthy controls was not significant compared to that of the patient group. It is possible that the healthy volunteers had similar disc pathologies as there is a high prevalence of disc pathologies in asymptomatic patients [36, 37] which could explain the lateral shift also in healthy subjects in the current study. However, whether the healthy volunteers were suffering from disc herniation was not systematically investigated by MRI or CT. The healthy controls were only investigated clinically. This should be taken into account in further research.

There are different methods available to measure LS, for example, the plumbline method and the shadow method [13, 14, 38]. The plumbline technique investigated by McLean et al. indicates that it is possible to measure a trunk list within a reliability of 4 mm [14]. In the present study, RS was chosen as the method of measurement for LS. RS is a radiation-free, noninvasive technique that has demonstrated very good inter- and intratester reliability as well [18, 21].

Fritz and Georg analysed LS in patients with acute and chronic pain [39]. The present study included patients with acute or chronic back pain as well as healthy volunteers. The stage of chronification measured by the MPSS has no influence on the posture or the measured parameters.

The patients demonstrated a significant different posture compared to the healthy subjects which was shown also by Khallaf [23]. In both studies the pelvis is increased lateral tilted in patients. The lordotic angle was increased in the patient's group of Khallaf. In contrast to Khallaf, our patients demonstrated a decreased lordotic angle which fits to the decreased anterior inclined pelvis.

In our study, there was a significant difference with respect to the BMI in both groups. Liljenqvist and colleagues reported that the thickness of soft tissue may result in measurement inaccuracies [40]. In contrast, Mohokum et al. did not find any differences in accuracy between subgroups with respect to BMI in a collective of young, healthy volunteers. Therefore, we did not assume any coherence of our results in relation to BMI. The differences in body weight or BMI were not caused by gender effect. 11 women and 28/25 men were examined in both groups. One reason of an increased body weight in patients could be that the patients were not so sporty and agile compared to the healthy subjects due to their pain.

The force of gravity affects bone positions and trunk muscle activities. In the supine or standing position, these parameters are different. In patients with low back pain, the onset of symptoms typically decreases in the supine position and increases in the standing position. Therefore, an RS investigation is more functional than MRI or CT, which is often performed in the horizontal position. Some researchers have investigated LS by radiography [35, 41]. One limitation of the present study is that there was no reference standard used for detecting LS, for instance, radiography.

RS can be applied to monitor postural changes, but caution should be taken when comparing absolute values because RS uses reference contours only. Radiological methods can derive the position of the spinous process directly from the morphology of internal bone structures.

Further research on this topic needs to be done. An investigation should be performed into other spine pathologies to find a specific pattern and to define demarcations between different pathologies.

## 5. Conclusions

The rasterstereography can identify changes of the posture of the spine in all three dimensions. This is the first study which illustrated the lateral shift using RS. Patients with disc herniation and low back pain show no increase in LS compared to healthy controls. Maybe, a lateral shift is more common in healthy patients as supposed. Alternatively, patients with disc herniation demonstrate other distinctive, significant parameters: an increased pelvic tilt, a decreased anterior pelvic inclination, a lower lordotic angle, and a higher amplitude of trunk torsion. In a further study, patients should be grouped, for example, based on their pathology like radiculopathy and discogenic pain to evaluate differences in posture.

## Figures and Tables

**Table 1:** Characteristics of participants.

**Parameter**	**Group**	**Mean**	**SD**	***p***
**Age (years)**	Patient group	48.2	9.4	.721
	Healthy controls	47.4	9.5	
**Height (cm)**	Patient group	175.1	10.8	.898
	Healthy controls	175.4	10.1	
**Weight (kg)**	Patient group	87.4	15.0	.014*∗*
	Healthy controls	79.3	12.6	
**BMI (kg/m** ^**2**^ **)**	Patient group	28.5	4.5	.002*∗∗*
	Healthy controls	25.7	2.9	

Comparison between patient group and healthy controls: mean, standard deviation (SD), and *p* value were illustrated for age, height, weight, and BMI.

*∗* Significance on the level of *p ≤*.05.

*∗∗* Significance on the level of *p* ≤ .01.

**Table 2:** Parameters of trunk, lumbar spine, and pelvis.

**Parameter**	**Group**	**Mean**	**SD**	***p***
**PT **[°]	Patient group	5.9	9.2	.016*∗*
	Healthy controls	2.0	2.4	
**PT [mm]**	Patient group	10.6	19.9	.033*∗*
	Healthy controls	3.3	3.7	
**PI (dimples) **[°]	Patient group	12.5	7.5	.002*∗∗*
	Healthy controls	17.8	6.9	
**PI (symmetry) **[°]	Patient group	15.0	10.2	.015*∗*
	Healthy controls	20.4	8.5	
**TT **[°]	Patient group	7.5	6.2	.017*∗*
	Healthy controls	4.5	4.1	
**LA **[°]	Patient group	27.5	9.6	.022*∗*
	Healthy controls	32.7	9.5	

Comparison between patient group and healthy controls: mean, standard deviation (SD), and *p* value were illustrated for PT [°], PT [mm], PT (dimples), PI (symmetry) [°], TT [°], and LA [°].

PT = pelvic tilt; PI (dimples) = pelvic inclination (dimples); PI (symmetry) = pelvic inclination in relation to the symmetry line; TT = trunk torsion; LA = lordotic angle T12 – DM

*∗* Significance on the level of *p* ≤ .05.

*∗∗* Significance on the level of *p* ≤ .01.

**Table 3:** Correlations with the FFbH-R.

**Parameter**	**Pearson r**	***p***
BMI	0.310	.007*∗∗*
MPSS	0.445	.005*∗∗*
Pain chart	0.643	≤ .001*∗∗*
NRS	0.804	≤ .001*∗∗*
Drug intake	0.545	≤ .001*∗∗*
PDI	0.793	≤ .001*∗∗*

BMI, MPSS, pain chart, NRS, drug intake, and PDI were correlated with the FFbH-R.

*∗* Significance on the level of *p* ≤ .05.

*∗∗* Significance on the level of *p≤*.01.

**Table 4:** Correlations with the PDI.

**Parameter**	**Pearson r**	***p***
Weight	0.246	.033*∗*
BMI	0.360	.002*∗∗*
MPSS	0.369	.021*∗*
Pain chart	0.623	≤ .001*∗∗*
NRS	0.785	≤ .001*∗∗*
Drug intake	0.379	≤ .001*∗∗*
FFbH-R	0.793	≤ .001*∗∗*

Weight, BMI, MPSS, pain chart, NRS, drug intake, and FFbH-R were correlated with the PDI.

*∗* Significance on the level of *p* ≤ .05.

*∗∗* Significance on the level of *p* ≤ .01.

## Data Availability

Data are available upon request.

## References

[1] Patrick N., Emanski E., Knaub M. A. (2014). Acute and chronic low back pain. *Medical Clinics of North America*.

[2] Schumacher J., Brähler E. (1999). The prevalence of pain in the German population: Results of population- based studies with the Giessen Subjective Complaints List (Giessener Beschwerdebogen GBB). *Der Schmerz*.

[3] Schmidt C. O., Kohlmann T. (2005). What do we know about the symptoms of back pain? Epidemiological results on prevalence, incidence, progression and risk factors. *Zeitschrift für Orthopädie und ihre Grenzgebiete*.

[4] Schmidt C. O., Raspe H., Pfingsten M. (2007). Back pain in the German adult population: Prevalence, severity, and sociodemographic correlates in a multiregional survey. *The Spine Journal*.

[5] Airaksinen O., Brox J. I., Cedraschi C. (2006). Chapter 4: European guidelines for the management of chronic nonspecific low back pain. *European Spine Journal*.

[6] Anderson P. A., McCormick P. C., Angevine P. D. (2008). Randomized controlled trials of the treatment of lumbar disk herniation: 1983-2007. *Journal of the American Academy of OrthopaedicSurgeons *.

[7] Heliovaara M., Impivaara O., Sievers K. (1987). Lumbar disc syndrome in Finland. *Journal of Epidemiology and Community Health*.

[8] Müller G. (2001). Problems of diagnostic assessment in low back patients. *Der Schmerz*.

[9] Stienen M. N., Cadosch D., Hildebrandt G., Gautschi O. P. (2011). The lumbar disc herniation - Management, clinical aspects and current recommendations. *Praxis*.

[10] Porter R. W., Miller C. G. (1986). Back pain and trunk list. *The Spine Journal*.

[11] McKenzie R. A., May S. (2003). *The Lumbar Spine. Mechanical Diagnosis and Therapy*.

[12] Gillan M. G. C., Ross J. C., McLean I. P., Porter R. W. (1998). The natural history of trunk list, its associated disability and the influence of McKenzie management. *European Spine Journal*.

[13] Donahue M. S., Riddle D. L., Sullivan M. S. (1996). Intertester reliability of a modified version of McKenzie's lateral shift assessments obtained on patients with low back pain. *Physical Therapy in Sport*.

[14] McLean I. P., Gillan M. G. C., Ross J. C., Aspden R. M., Porter R. W. (1996). A comparison of methods for measuring trunk list: A simple plumbline is the best. *The Spine Journal*.

[15] Drerup B., Hierholzer E. (1994). Back shape measurement using video rasterstereography and three-dimensional reconstruction of spinal shape. *Clinical Biomechanics*.

[16] Hackenberg L., Hierholzer E., Pötzl W., Götze C., Liljenqvist U. (2003). Rasterstereographic back shape analysis in idiopathic scoliosis after posterior correction and fusion. *Clinical Biomechanics*.

[17] Hackenberg L., Hierholzer E., Pötzl W., Götze C., Liljenqvist U. (2003). Rasterstereographic back shape analysis in idiopathic scoliosis after anterior correction and fusion. *Clinical Biomechanics*.

[18] Mohokum M., Mendoza S., Udo W., Sitter H., Paletta J. R., Skwara A. (2010). Reproducibility of rasterstereography for kyphotic and lordotic angles, trunk length, and trunk inclination: a reliability study. *Spine (Phila Pa 1976)*.

[19] Mohokum M., Schülein S., Skwara A. (2015). The validity of rasterstereography: A systematic review. *Orthopedic Reviews*.

[20] Crawford R. J., Price R. I., Singer K. P. (2009). The effect of interspinous implant surgery on back surface shape and radiographic lumbar curvature. *Clinical Biomechanics*.

[21] Schülein S., Mendoza S., Malzkorn R., Harms J., Skwara A. (2013). Rasterstereographic evaluation of interobserver and intraobserver reliability in postsurgical adolescent idiopathic scoliosis patients. *Journal of Spinal Disorders & Techniques*.

[22] Tabard-Fougère A., Bonnefoy-Mazure A., Hanquinet S., Lascombes P., Armand S., Dayer R. (2017). Validity and reliability of spine rasterstereography in patients with adolescent idiopathic scoliosis. *The Spine Journal*.

[23] Khallaf M. E. (2017). Three dimensional analysis of spino-pelvic alignment in individuals with acutely herniated lumbar intervertebral disc. *Journal of Back and Musculoskeletal Rehabilitation*.

[24] Lippold C., Moiseenko T., Drerup B., Schilgen M., Végh A., Danesh G. (2012). Spine deviations and orthodontic treatment of asymmetric malocclusions in children. *BMC Musculoskeletal Disorders*.

[25] Hawker G. A., Mian S., Kendzerska T., French M. (2011). Measures of adult pain: Visual Analog Scale for Pain (VAS Pain), Numeric Rating Scale for Pain (NRS Pain), McGill Pain Questionnaire (MPQ), Short-Form McGill Pain Questionnaire (SF-MPQ), Chronic Pain Grade Scale (CPGS), Short Form-36 Bodily Pain Scale (SF-36 BPS), and measure of Intermittent and Constant Osteoarthritis Pain (ICOAP). *Arthritis Care & Research*.

[26] Kohlmann T., Raspe H. (1996). Hannover Functional Questionnaire in ambulatory diagnosis of functional disability caused by backache. *Rehabilitation (Stuttg)*.

[27] Gronblad M., Hupli M., Wennerstrand P. (1993). Intercorrelation and test-retest reliability of the Pain Disability Index (PDI) and the Oswestry Disability Questionnaire (ODQ) and their correlation with pain intensity in low back pain patients. *The Clinical Journal of Pain*.

[28] Hall T., Cacho A., McNee C., Riches J., Walsh J. (2001). Effects of the mulligan traction straight leg raise technique on range of movement. *Journal of Manual & Manipulative Therapy*.

[29] Drerup B. (2014). Rasterstereographic measurement of scoliotic deformity. *Scoliosis*.

[30] Drerup B., Hierholzer E. (1987). Automatic localization of anatomical landmarks on the back surface and construction of a body-fixed coordinate system. *Journal of Biomechanics*.

[31] Drerup B., Hierholzer E. (1985). Objective determination of anatomical landmarks on the body surface: Measurement of the vertebra prominens from surface curvature. *Journal of Biomechanics*.

[32] Degenhardt B., Starks Z., Bhatia S., Franklin G.-A. (2017). Appraisal of the DIERS method for calculating postural measurements: An observational study. *Scoliosis and Spinal Disorders*.

[33] Negrini S., Donzelli S., Aulisa A. G. (2018). 2016 SOSORT guidelines: Orthopaedic and rehabilitation treatment of idiopathic scoliosis during growth. *Scoliosis and Spinal Disorders*.

[34] Harrison D. E., Cailliet R., Harrison D. D., Janik T. J., Troyanovich S. J., Coleman R. R. (1999). Lumbar coupling during lateral translations of the thoracic cage relative to a fixed pelvis. *Clinical Biomechanics*.

[35] Harrison D. E., Cailliet R., Betz J. W. (2005). A non-randomized clinical control trial of Harrison mirror image methods for correcting trunk list (lateral translations of the thoracic cage) in patients with chronic low back pain. *European Spine Journal*.

[36] Stadnik T. W., Lee R. R., Coen H. L., Neirynck E. C., Buisseret T. S., Osteaux M. J. C. (1998). Annular tears and disk herniation: prevalence and contrast enhancement on MR images in the absence of low back pain or sciatica. *Radiology*.

[37] Ernst C. W., Stadnik T. W., Peeters E., Breucq C., Osteaux M. J. C. (2005). Prevalence of annular tears and disc herniations on MR images of the cervical spine in symptom free volunteers. *European Journal of Radiology*.

[38] Razmjou H., Kramer J. F., Yamada R. (2000). Intertester reliability of the McKenzie evaluation in assessing patients with mechanical low-back pain. *Journal of Orthopaedic & Sports Physical Therapy*.

[39] Fritz J. M., George S. (2000). The use of a classification approach to identify subgroups of patients with acute low back pain: Interrater reliability and short-term treatment outcomes. *The Spine Journal*.

[40] Liljenqvist U., Halm H., Hierholzer E., Drerup B., Weiland M. (1998). Three-dimensional surface measurement of spinal deformities using video rasterstereography. *Zeitschrift für Orthopädie und ihre Grenzgebiete*.

[41] Arangio G. A., Hartzell S. M., Reed J. F. (1990). Significance of lumbosacral list and low-back pain: A controlled radiographic study. *The Spine Journal*.

